# Lipid-lowering therapy for primary prevention of cardiovascular disease in adults aged 75 years and older: a narrative review

**DOI:** 10.1016/j.ijcrp.2026.200646

**Published:** 2026-04-28

**Authors:** Dominique Stephan, Camille Zamperini, Elena-Mihaela Cordeanu

**Affiliations:** Hypertension and Vascular Diseases Unit, University Hospital Strasbourg, France

**Keywords:** Primary prevention, Lipid-lowering therapy, Older adults

## Abstract

**Background:**

Adults aged 75 years and older carry the highest absolute risk of cardiovascular disease (CVD) yet remain substantially underrepresented in randomized trials evaluating statin therapy for primary prevention. This evidence gap creates uncertainty regarding the appropriateness of initiating lipid-lowering therapy in this population.

**Methods:**

We conducted a narrative review of randomized controlled trials, meta-analyses, and large observational studies evaluating statin therapy for primary CVD prevention in adults aged 75 years and older. We searched PubMed and reference lists of relevant articles through December 2024.

**Results:**

Direct randomized evidence for primary prevention in adults over 75 years is limited. The PROSPER trial included a mixed population of primary and secondary prevention patients aged 70–82 years. Post-hoc analyses of ALLHAT-LLT showed no benefit and a trend toward harm in patients aged 75 years and older. Meta-analyses of observational data suggest mortality reductions of 12–14% associated with statin use, though subject to confounding. The time to benefit for cardiovascular event reduction is approximately 2.5 years, which must be weighed against individual life expectancy.

**Conclusions:**

Current evidence does not support routine statin initiation for primary prevention in all adults over 75 years. Treatment decisions should be individualized based on cardiovascular risk, life expectancy, frailty status, patient preferences, and competing health priorities. Results from the STAREE and PREVENTABLE trials, expected in 2025–2027, will provide definitive guidance.

## Highlights

• Randomized evidence for statins in primary prevention >75 years remains limited.
• Observational studies suggest benefit but are prone to residual confounding.
• Time to benefit (∼2.5 years) is critical when life expectancy is limited.
• Statin decisions in older adults should be individualized and preference-based.
• Ongoing trials (STAREE, PREVENTABLE) will provide definitive guidance.

## Graphical abstract

**Figure:**
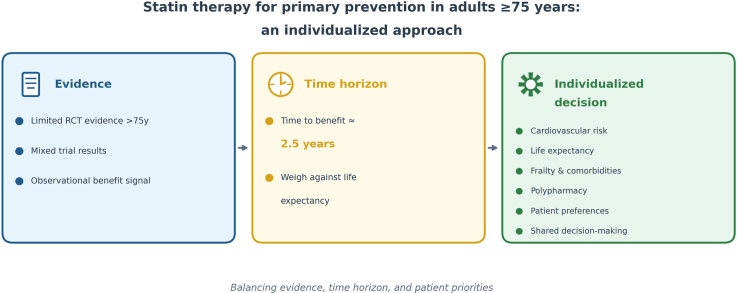

## 1 Introduction

Cardiovascular disease remains the leading cause of mortality worldwide, with its burden increasing substantially with advancing age. Adults aged 75 years and older represent a rapidly growing population segment in aging societies and carry the highest absolute risk of atherosclerotic cardiovascular disease, reflecting cumulative lifetime exposure to vascular risk factors and arterial aging. Despite advanced chronological age, life expectancy and functional status remain highly variable in this group, making cardiovascular prevention potentially relevant in selected individuals. However, pronounced biological heterogeneity, competing risks, and the high prevalence of frailty complicate the application of uniform preventive strategies [1,2]. Statins have consistently reduced major cardiovascular events in randomized trials. However, older adults, particularly those aged over 75 years, have been markedly underrepresented. This evidence gap has led US and European guidelines to refrain from recommending routine statin therapy for primary prevention in this age group [3,4]. The 2018 American College of Cardiology/American Heart Association guideline states that initiation of statin therapy in individuals older than 75 years may be considered after clinician–patient discussion, explicitly acknowledging the limited randomized evidence [3]. Similarly, the 2021 European Society of Cardiology guidelines provide only a Class IIb recommendation for lipid-lowering therapy in older patients at very high cardiovascular risk, emphasizing shared decision-making [4]. A critical consideration often overlooked in clinical practice is the concept of "time to benefit", the duration of treatment required before meaningful clinical benefit accrues. A recent meta-analysis estimated that approximately 2.5 years of statin treatment are needed to prevent one major adverse cardiovascular event per 100 patients treated [5]. This lag time must be weighed against individual life expectancy when considering statin initiation in older adults.

An additional complexity specific to this age group is the evolving relationship between LDL cholesterol (LDL-C) levels and cardiovascular risk at advanced age. While the causal role of LDL-C in atherosclerosis is well established across the lifespan, epidemiological data suggest that the association between LDL-C and all-cause mortality may weaken or even reverse at very old age, a phenomenon sometimes termed the "LDL paradox" in the elderly [6]. This may partly reflect reverse causation, as low LDL-C in older adults can be a marker of malnutrition, chronic inflammation, or occult malignancy rather than a treatment effect [6]. Conversely, Mendelian randomization studies support a lifelong causal effect of LDL-C on atherosclerotic risk independent of age [7]. The clinical implication is that LDL-C values should not be used in isolation to drive treatment decisions in adults aged 75 years and older, rather absolute cardiovascular risk, comorbidity burden, and functional status must be integrated into the clinical assessment. Furthermore, current risk calculators such as SCORE2-OP may underestimate or overestimate risk in specific elderly phenotypes, particularly those with sarcopenia, cachexia, or multimorbidity [8].

Therefore, the aim of this narrative review is to summarize and critically appraise the available evidence on lipid-lowering therapy for primary cardiovascular prevention in adults aged 75 years and older, with particular attention to the balance between expected benefit, time to benefit, and life expectancy.

## 2 Evidence from randomized controlled trials

The evidence from randomized controlled trials specifically examining statin use for primary prevention in adults over 75 years is remarkably sparse. Most available data come from subgroup analyses of larger trials that were not primarily designed to address this question (Table 1). The PROSPER (Prospective Study of Pravastatin in the Elderly at Risk) trial remains the only large-scale randomized trial specifically designed to evaluate statin therapy in elderly patients. This study enrolled 5804 men and women aged 70 to 82 years with either pre-existing vascular disease or elevated cardiovascular risk [9]. After a mean follow-up of 3.2 years, pravastatin 40 mg reduced the composite primary endpoint of coronary death, non-fatal myocardial infarction, and stroke by 15% (hazard ratio 0.85, 95% CI 0.74–0.97). However, approximately 44% of participants had established CVD at baseline, limiting the applicability of these findings to primary prevention. Subgroup and extended follow-up analyses indicated that the observed benefit was largely confined to participants with pre-existing vascular disease, with no significant effect on stroke or all-cause mortality among those treated for primary prevention [10].

**Table 1:** Randomized controlled trials of statin therapy in older adults.

Study	Year	Design	N	Age (y)	F/U	Intervention	Endpoint	HR (95% CI)
PROSPER	2002	RCT, DB	5804	70-82	3.2 y	Pravastatin 40 mg	CV death, MI, stroke	0.85 (0.74-0.97)^a^
PROSPER (1° prev)	2002	Subgroup	3239	70-82	3.2 y	Pravastatin 40 mg	CV death, MI, stroke	0.94 (0.77-1.15) NS
ALLHAT-LLT (≥65y)	2017	RCT, OL	2867	≥65	6.0 y	Pravastatin 40 mg	All-cause mortality	1.08 (0.85-1.37) NS
ALLHAT-LLT (≥75y)	2017	Post-hoc	1467	≥75	6.0 y	Pravastatin 40 mg	All-cause mortality	1.34 (0.98-1.84) NS
JUPITER (≥70y)	2008	RCT, DB	5695	≥70	1.9 y	Rosuvastatin 20 mg	MI, stroke, CV death	0.61 (0.46-0.82)^a^
HOPE-3 (≥70y)	2016	RCT, DB	3086	≥70	5.6 y	Rosuvastatin 10 mg	MI, stroke, CV death	0.83 (0.64-1.07) NS

a Statistically significant. DB: double-blind; OL: open-label; NS: not significant; CV: cardiovascular; MI: myocardial infarction; 1°: primary prevention.

The ALLHAT-LLT (Antihypertensive and Lipid-Lowering Treatment to Prevent Heart Attack Trial) compared pravastatin with usual care in hypertensive patients with moderate hypercholesterolemia. A post-hoc analysis of participants aged 65 years and older in primary prevention found no significant benefit of pravastatin on all-cause mortality (HR 1.08, 95% CI 0.85–1.37). More concerning, among patients aged 75 years and older, there was a non-significant trend toward increased mortality (HR 1.34, 95% CI 0.98–1.84) [11]. These results must be interpreted cautiously given the open-label design and substantial cross-over between treatment groups (78% of pravastatin patients still on treatment versus 29% of usual care patients taking statins at 6 years).

Ridker et al. performed a meta-analysis of age-specific outcome data from two contemporary primary prevention statin trials, JUPITER and HOPE-3, that included substantial proportions of older adults [12]. Among 8781 participants aged 70 years or older, rosuvastatin therapy was associated with a 26% reduction in the composite endpoint of non-fatal myocardial infarction, non-fatal stroke, or cardiovascular death (HR 0.74, 95% CI 0.61–0.91). However, no significant reduction in all-cause mortality was observed, and the applicability to those aged 75 years and older specifically remains uncertain given the small numbers in this subgroup.

The Cholesterol Treatment Trialists' (CTT) Collaboration conducted a landmark individual participant data meta-analysis of 28 randomized trials involving 186,854 participants [13]. The analysis found that statin therapy produced significant reductions in major vascular events across all age groups, including those older than 75 years. However, among primary prevention patients over 75 years, the benefit was less clear (RR 0.92, 95% CI 0.73–1.16), with wider confidence intervals and the authors acknowledging insufficient direct evidence in this subgroup. Notably, using Bayesian analysis, Kostis et al. found a statistically significant mortality reduction, suggesting persistent uncertainty rather than definitive absence of benefit [14]. It should be noted that the CTT meta-analysis included a large proportion of participants with established cardiovascular disease, and the primary prevention subgroup aged over 75 years was small, resulting in wide confidence intervals (RR 0.92, 95% CI 0.73-1.16) [13]. These findings should therefore be interpreted as indirect evidence, with limited direct applicability to the strictly primary prevention setting in the oldest age group. Similarly, the Ridker meta-analysis, while restricted to primary prevention, included participants primarily aged 70-75 years, limiting extrapolation to the over-75 subgroup specifically [12].

## 3 Evidence from observational studies

Given the limitations of randomized trial data, observational studies have attempted to fill the evidence gap, though they are inherently subject to confounding and healthy-user bias (Table 2). A systematic review and meta-analysis of observational studies by Awad et al., including over 800,000 participants, found that statin use in primary prevention patients aged 65 years and older was associated with a 14% reduction in all-cause mortality (HR 0.86, 95% CI 0.79–0.93) and a 20% reduction in CVD mortality [15]. Importantly, the beneficial association persisted in those over 75 years (HR 0.88, 95% CI 0.81–0.96). The Three-City cohort study by Alpérovitch et al. examined 7484 French adults aged 65 years and older without prior cardiovascular events [16]. After 9.1 years of follow-up, lipid-lowering drug users had a significantly reduced risk of ischemic stroke (HR 0.66, 95% CI 0.49–0.90) but no benefit for coronary heart disease events (HR 1.12, 95% CI 0.90–1.40). These findings highlight the heterogeneous effects of statins on different cardiovascular endpoints in older adults populations. In a population-based cohort study from Catalonia, Ramos et al. evaluated statin use for primary prevention in adults aged 75 years and older [17]. In individuals without diabetes, statin therapy was not associated with reductions in atherosclerotic events or all-cause mortality. In contrast, statin use was associated with lower cardiovascular events and mortality in patients aged 75–84 years with diabetes, suggesting that baseline cardiometabolic risk strongly modifies treatment benefit. Orkaby et al. investigated statin use in a large cohort of U.S. veterans aged 75 years and older without documented cardiovascular disease [18]. Statin initiation was associated with lower all-cause (HR 0.75, 95% CI 0.74–0.76) and cardiovascular mortality. However, this predominantly male population was subject to healthy-user and confounding-by-indication biases. A recent target trial emulation study from Hong Kong by Xu et al., published in 2024, compared statin initiation versus no initiation in adults aged 65 years and older, with separate analyses for those aged 75–84 years and 85 years and older [19]. Statin initiation was associated with reduced cardiovascular events in both age groups, including the very elderly, providing contemporary real-world evidence. However, as with all observational studies, residual confounding cannot be excluded.

**Table 2:** Key observational studies of statin therapy for primary prevention in older adults.

Study	Year	Country	Design	N	Age	F/U	Main Findings	Limitations
**Alpérovitch (3C)**	2015	France	Prospective cohort	7484	≥65y	9.1 y	Stroke HR 0.66 (0.49-0.90)^a^; CHD HR 1.12 NS	No coronary benefit
**Ramos et al.**	2018	Spain	Retrospective	46,864	≥75y	5.6 y	No benefit w/o DM; benefit with DM (75-84y)	Effect modif. by DM
**Orkaby (VA)**	2020	USA	Retrospective	326,981	≥75y	6.8 y	All-cause mortality HR 0.75 (0.74-0.76)^a^	Male; healthy user bias
**Bezin et al.**	2019	France	Retrospective	7882	≥75y	4.4 y	Benefit only with CV risk factors	Selected population
**Xu et al.**	2024	Hong Kong	Target trial emul.	42,680	≥65y	5.0 y	CV events ↓ in 75-84y and ≥85y	Residual confounding

a Statistically significant. CHD: coronary heart disease; DM: diabetes mellitus; CV: cardiovascular; VA: Veterans Affairs; 3C: Three-City Study.

Several methodological considerations limit the interpretation of observational evidence in this field. Despite advances in study design, including target trial emulation as used by Xu et al., residual confounding remains a fundamental concern [19,20]. Healthy-user bias, whereby patients who initiate statins tend to have better overall health behaviours, higher socioeconomic status, and more regular healthcare engagement, may substantially inflate observed mortality benefits [15]. Furthermore, the heterogeneity of findings across subgroups warrants attention: observational studies consistently suggest greater benefit in older adults with diabetes or established cardiometabolic risk factors [15], whereas benefit in non-diabetic or low-risk elderly patients appears attenuated or absent, as demonstrated by Ramos et al. [17]. This differential effect likely reflects the higher baseline absolute risk in diabetic patients, which amplifies any relative risk reduction into a clinically meaningful absolute benefit [15]. In low-risk, non-diabetic elderly patients, the same relative reduction translates into a smaller absolute benefit, which may be offset by treatment burden and adverse effects. These findings reinforce the need for risk-stratified rather than age-based prescribing decisions.

A synthesis of randomized and observational meta-analyses assessing statin therapy for primary prevention in older adults is presented in Table 3.

**Table 3:** Meta-analyses of statin therapy for primary prevention in older adults.

Meta-analysis	Year	Type	Studies/N	Age	Outcome	Effect (95% CI)	Interpretation
**CTT Collaboration**	2019	IPD MA (RCTs)	28 trials, n = 186,854	>75y, 1° prev	Major vascular events	RR 0.92 (0.73-1.16)	NS; insufficient evidence
**Ridker (JUPITER + HOPE-3)**	2017	IPD MA (RCTs)	2 trials, n = 8781	≥70y	MI, stroke, CV death	HR 0.74 (0.61-0.91)^a^	Significant benefit
**Kostis et al.**	2020	Aggregate MA	4 trials, n = 10,456	>75y, 1° prev	All-cause mortality	HR 0.86 (0.71-1.04)	NS freq.; sig. Bayesian
**Awad et al.**	2021	MA (Obs)	10 studies, n = 815,667	>75y	All-cause mortality	HR 0.88 (0.81-0.96)^a^	Significant (obs.)
**Teng et al.**	2015	MA (RCTs)	8 trials, n = 25,952	≥65y	MACE	RR 0.82 (0.74-0.92)^a^	Significant; limited >75y

a Statistically significant. IPD: individual participant data; MA: meta-analysis; Obs: observational; NS: not significant; freq.: frequentist; MACE: major adverse cardiovascular events.

## 4 Time to benefit and life expectancy considerations

A critical but often underappreciated consideration in prescribing preventive medications to older adults is the time required for treatment benefit to accrue relative to the patient's life expectancy. A pragmatic framework integrating estimated life expectancy and time to benefit is presented in Table 4. Yourman et al. conducted a survival meta-analysis of eight randomized trials including 65,383 adults aged 50–75 years without known CVD [5]. The study estimated that 2.5 years (95% CI 1.7–3.4) of statin treatment were needed to prevent one major adverse cardiovascular event per 100 patients treated. Notably, only one of eight trials demonstrated a mortality benefit. These findings suggest that statins are most appropriate for primary prevention in adults with a life expectancy exceeding 2.5 years. For patients aged 75 years and older, median life expectancy varies substantially based on health status: approximately 12 years for a healthy 75-year-old woman but less than 5 years for a frail individual with multiple comorbidities [21]. This heterogeneity underscores the importance of individualized assessment rather than age-based treatment thresholds. For patients with limited life expectancy due to serious illness or advanced frailty, the lag time to benefit may exceed remaining lifespan, making statin initiation inappropriate.

**Table 4:** Time to benefit vs. life expectancy: framework for clinical decision-making.

Age (y)	Health Status	Life Expectancy	Time to Benefit	Net Benefit ?	Clinical Implication
**75**	Healthy (top quartile)	14-16 years	2.5 years	**Yes**	Consider statin if high CV risk
**75**	Average health	10-12 years	2.5 years	**Likely yes**	Discuss benefits/risks; shared decision
**75**	Poor health (bottom quartile)	5-7 years	2.5 years	**Uncertain**	Weigh competing risks; consider deferring
**80**	Healthy (top quartile)	10-12 years	2.5 years	**Likely yes**	Consider statin if high CV risk
**80**	Average health	7-9 years	2.5 years	**Uncertain**	Individualize; consider frailty
**80**	Poor health (bottom quartile)	3-5 years	2.5 years	**Unlikely**	Deprescribing may be appropriate
**85**	Healthy (top quartile)	7-8 years	2.5 years	**Possible**	Very individualized decision
**85**	Average/poor health	3-5 years	2.5 years	**Unlikely**	Focus on symptom management

Life expectancy estimates based on ePrognosis indices. Time to benefit of 2.5 years based on Yourman et al. JAMA Intern Med 2021.

## 5 Statin discontinuation in older adults

While much attention focuses on initiating statin therapy, the question of whether to discontinue statins in older adults already receiving treatment is equally relevant, particularly in the context of polypharmacy, declining health, or approaching end of life. Giral et al. examined the cardiovascular effect of discontinuing statins at age 75 years in a French nationwide cohort of primary prevention patients [22]. Statin discontinuation was associated with a 33% increased risk of hospital admission for cardiovascular events (HR 1.33, 95% CI 1.18–1.50) compared with continuation. A Danish registry study by Thompson et al. found that among adults aged 75 years and older who had been treated with statins for at least 5 years, discontinuation was associated with a 32% increased risk of major adverse cardiovascular events in primary prevention (HR 1.32, 95% CI 1.18–1.48) [23]. A systematic review by Peixoto et al. examining 36 studies on statin discontinuation concluded that stopping statins in patients not approaching end of life was associated with nearly 2-fold increased all-cause mortality, 63% increased cardiovascular mortality, and 31% increased cardiovascular events [24]. However, in a small randomized trial of patients with life expectancy less than one year, statin discontinuation improved quality of life without significantly affecting 60-day or 1-year mortality. It is important to note that the discontinuation studies by Giral et al. [22], Thompson et al. [23], and the systematic review by Peixoto et al. [24] included substantial proportions of patients with prior cardiovascular events. The applicability of these findings to primary prevention-only patients aged 75 years and older is therefore uncertain, and the magnitude of risk associated with discontinuation may differ in this specific subgroup. These findings suggest caution when considering statin discontinuation in older adults receiving long-term therapy for primary prevention, except in the context of limited life expectancy, significant adverse effects, or patient preference prioritizing quality of life over cardiovascular risk reduction.

## 6 Safety considerations

Elderly patients may be more susceptible to statin-related adverse effects due to polypharmacy, altered pharmacokinetics, and increased frailty. Muscle-related symptoms, including myalgia and rarely rhabdomyolysis, are the most commonly reported adverse effects and may contribute to medication discontinuation [25]. Meta-analyses have generally not shown significantly increased rates of myopathy or serious adverse events in elderly patients compared with younger populations, although reporting may be incomplete. The potential association between statins and cognitive impairment has been a particular concern in elderly populations. However, the PROSPER trial found no significant effect of pravastatin on cognitive function over 3.2 years of follow-up [9]. A Cochrane review and multiple meta-analyses have not demonstrated cognitive harm from statin therapy [26]. New-onset diabetes is an established adverse effect of statin therapy, with meta-analyses suggesting approximately 9% increased relative risk [27]. In elderly patients who may already have impaired glucose tolerance, this effect warrants consideration, though the absolute cardiovascular benefit likely outweighs the diabetes risk in high-risk individuals.

## 7 Frailty, nutritional status and lipid levels

Frailty deserves specific attention not only as a determinant of life expectancy and treatment tolerance, but also as a potential modifier of baseline lipid profiles [21,28]. Frail older adults frequently exhibit reduced dietary intake, impaired nutritional absorption, and unintentional weight loss, all of which may independently lower circulating LDL-C levels [6,29]. As a result, frail patients may appear to have favourable lipid profiles without reflecting a biologically protective state. In this context, low LDL-C in a frail individual may be a marker of nutritional vulnerability rather than reduced atherosclerotic burden, and should prompt clinical reassessment rather than therapeutic reassurance [6]. Conversely, the initiation of statins in frail patients with already low LDL-C raises questions about the magnitude of achievable LDL-C reduction and the expected absolute cardiovascular benefit. Frailty is also associated with increased susceptibility to adverse drug effects: myopathy risk may be amplified by low muscle mass, impaired hepatic and renal drug clearance, and polypharmacy-driven pharmacokinetic interactions [25,30]. Formal frailty assessment, using validated instruments such as the Fried phenotype [28], the Clinical Frailty Scale [31], or the FRAIL questionnaire [32], should therefore be an integral component of the shared decision-making process when considering statin initiation or continuation in adults aged 75 years and older. Current guidelines lack specific LDL-C targets for frail elderly patients in primary prevention, highlighting a critical evidence gap [3,4].

## 8 Non-statin lipid-lowering therapies in older adults

Although statins remain the cornerstone of lipid-lowering therapy for cardiovascular prevention, non-statin agents including ezetimibe and proprotein convertase subtilisin/kexin type 9 (PCSK9) inhibitors have transformed secondary prevention practice over the past decade. However, evidence specifically supporting their use in primary prevention among adults aged 75 years and older is virtually absent.

Ezetimibe, an intestinal cholesterol absorption inhibitor, demonstrated incremental cardiovascular benefit when added to statin therapy in the IMPROVE-IT trial, but this trial enrolled a secondary prevention population with a mean age of 64 years, limiting direct applicability [33]. No dedicated primary prevention trial of ezetimibe has been conducted in elderly adults. In frail or polymedicated patients who are statin-intolerant, ezetimibe represents a low-interaction, generally well-tolerated alternative, but its absolute benefit in primary prevention remains unquantified in this age group.

PCSK9 inhibitors (evolocumab, alirocumab) have demonstrated robust LDL-C lowering and cardiovascular event reduction in secondary prevention trials (FOURIER, ODYSSEY OUTCOMES), including in older subgroups [34,35]. However, primary prevention data are lacking, cost-effectiveness at advanced age is uncertain, and the injectable route of administration may pose adherence challenges in frail or cognitively impaired patients. Furthermore, the long-term safety implications of very low LDL-C levels, including potential effects on cognitive function, have not been specifically studied in the oldest primary prevention population [[36], [37], [38]]. Inclisiran, a small interfering RNA agent administered twice yearly, offers potential advantages in terms of adherence in elderly patients, but primary prevention data in adults over 75 years do not yet exist [39].

Until dedicated trials address these questions, non-statin therapies in primary prevention among older adults should be considered on an individualized basis, reserved for patients with statin intolerance and high absolute cardiovascular risk, and prescribed within a shared decision-making framework.

## 9 Ongoing clinical trials

**Fig. 1:**
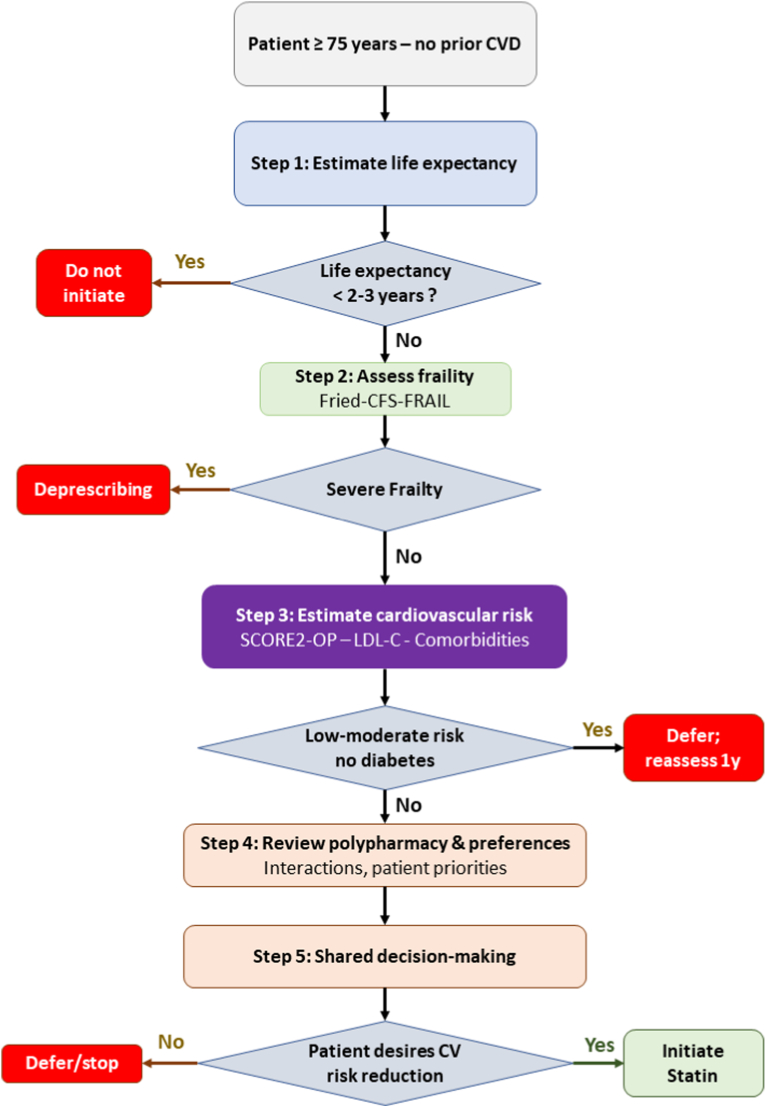
Proposed algorithm for individualized statin therapy decision-making in primary cardiovascular prevention in adults aged ≥75 years. CFS: Clinical Frailty Scale; SCORE2-OP: Systematic Coronary Risk Evaluation 2 – Older Persons; CV: cardiovascular.

Two large-scale randomized controlled trials are currently underway to definitively address the question of statin therapy for primary prevention in elderly populations. The STAREE (STAtins in Reducing Events in the Elderly) trial is an Australian randomized, double-blind, placebo-controlled trial that has enrolled 9971 community-dwelling adults aged 70 years and older without prior CVD, diabetes, or dementia [40]. Participants are randomized to atorvastatin 40 mg daily or placebo, with co-primary endpoints of disability-free survival and major cardiovascular events. Results are anticipated in late 2025. The PREVENTABLE (Pragmatic Evaluation of Events and Benefits of Lipid-lowering in Older Adults) trial aims to enroll 20,000 US adults aged 75 years and older, comparing atorvastatin 40 mg with placebo [41]. The primary outcome is a composite of death, dementia, and persistent disability over four years. This trial specifically targets the very elderly population and will provide crucial evidence for clinical decision-making, with results expected around 2027. Given the heterogeneity of health status and cardiovascular risk among adults aged 75 years and older, treatment decisions should be individualized. Key clinical factors that may favor or argue against statin initiation for primary prevention are summarized in Table 5. A proposed stepwise algorithm integrating these factors into a structured decision-making framework is presented in Fig. 1.

**Table 5:** Factors to consider when deciding on statin therapy for primary prevention in adults ≥75 years.

Factor	Favors Statin Initiation	Favors Deferring/Not Initiating	Assessment Tool
**Life expectancy**	>5 years	<2-3 years	ePrognosis calculators; clinical judgment
**Cardiovascular risk**	High (≥20% 10-year risk) or very high risk factors	Low-moderate risk; no diabetes	SCORE2-OP; risk calculators
**Functional status**	Independent; robust	Frail; dependent; nursing home resident	Frailty indices; ADL assessment
**Comorbidities**	Few; well-controlled	Multiple; life-limiting illness; cancer	Charlson index; clinical assessment
**Polypharmacy**	Few medications; low interaction risk	≥10 medications; high interaction risk	Medication review; BEERS criteria
**Cognitive status**	Intact cognition	Dementia; difficulty with adherence	MMSE; MoCA; clinical assessment
**Patient preference**	Desires CV risk reduction	Prioritizes QoL; avoids medications	Shared decision-making discussion
**Prior statin use**	Tolerating well; on therapy >2 years	Prior intolerance; myalgias	Symptom review; rechallenge if needed

ADL: activities of daily living; SCORE2-OP: Systematic COronary Risk Evaluation 2 - Older Persons; QoL: quality of life; MMSE: Mini-Mental State Examination; MoCA: Montreal Cognitive Assessment; CV: cardiovascular.

## 10 Conclusion

The current evidence regarding lipid-lowering therapy for primary prevention of cardiovascular disease in adults aged 75 years and older remains inconclusive. While observational studies generally suggest benefit, particularly in high-risk subgroups such as those with diabetes, the lack of robust randomized trial data specifically in this population creates persistent uncertainty. The time to benefit of approximately 2.5 years for cardiovascular event reduction must be weighed against individual life expectancy and competing health priorities. Treatment decisions should be individualized through shared decision-making, considering cardiovascular risk burden, estimated life expectancy, frailty status, potential for drug interactions, polypharmacy burden, and patient preferences regarding medication use (Fig. 1). For healthy older adults with elevated cardiovascular risk and reasonable life expectancy exceeding 3–5 years, statin therapy may provide meaningful benefit. Conversely, for frail individuals with limited life expectancy, multiple comorbidities, or those prioritizing quality of life, deferring statin initiation or discontinuing existing therapy may be appropriate. The STAREE and PREVENTABLE trials are expected to provide definitive guidance and should inform future guideline recommendations. Until their results are available, clinicians should resist both therapeutic nihilism and reflexive prescribing, instead engaging each patient in thoughtful discussion of the uncertain benefits and potential risks of statin therapy for primary prevention in advanced age.

## Declaration of generative AI and AI-assisted technologies

During the preparation of this work, the authors used Claude and ChatGPT to support language editing and improve clarity and structure of the manuscript. After using these tools, the authors critically reviewed, edited, and validated all content and take full responsibility for the accuracy and integrity of the published article.

## Funding

This research did not receive any specific grant from funding agencies in the public, commercial, or not-for-profit sectors.

## CRediT authorship contribution statement

**Dominique Stephan:** Conceptualization, Methodology, Writing – original draft, Writing – review & editing. **Camille Zamperini:** Data curation, Formal analysis, Methodology, Validation. **Elena-Mihaela Cordeanu:** Conceptualization, Data curation, Formal analysis, Methodology, Validation, Writing – original draft.

## Declaration of competing interest

None.
